# A nationwide survey on health resources and clinical practices during the early COVID-19 pandemic in Brazil

**DOI:** 10.5935/0103-507X.20220005-en

**Published:** 2022

**Authors:** Pedro Paulo Zanella do Amaral Campos, Guilherme Martins de Souza, Thais Midega, Hélio Penna Guimarães, Thiago Domingos Corrêa, Ricardo Luiz Cordioli

**Affiliations:** 1 Hospital Israelita Albert Einstein - São Paulo (SP), Brazil.

**Keywords:** COVID-19, Coronavirus infections, Pandemics, Emergency service, hospital, Practice patterns, physicians, Hospital administration, Surveys and questionnaires, Intensive care units, Brazil

## Abstract

**Objective:**

To evaluate clinical practices and hospital resource organization during the early COVID-19 pandemic in Brazil.

**Methods:**

This was a multicenter, cross-sectional survey. An electronic questionnaire was provided to emergency department and intensive care unit physicians attending COVID-19 patients. The survey comprised four domains: characteristics of the participants, clinical practices, COVID-19 treatment protocols and hospital resource organization.

**Results:**

Between May and June 2020, 284 participants [median (interquartile ranges) age 39 (33 - 47) years, 56.3% men] responded to the survey; 33% were intensivists, and 9% were emergency medicine specialists. Half of the respondents worked in public hospitals. Noninvasive ventilation (89% *versus* 73%; p = 0.001) and highflow nasal cannula (49% *versus* 32%; p = 0.005) were reported to be more commonly available in private hospitals than in public hospitals. Mechanical ventilation was more commonly used in public hospitals than private hospitals (70% *versus* 50%; p = 0,024). In the Emergency Departments, positive endexpiratory pressure was most commonly adjusted according to SpO_2,_ while in the intensive care units, positive end-expiratory pressure was adjusted according to the best lung compliance. In the Emergency Departments, 25% of the respondents did not know how to set positive end-expiratory pressure. Compared to private hospitals, public hospitals had a lower availability of protocols for personal protection equipment during tracheal intubation (82% *versus* 94%; p = 0.005), managing mechanical ventilation [64% *versus* 75%; p = 0.006] and weaning patients from mechanical ventilation [34% *versus* 54%; p = 0.002]. Finally, patients spent less time in the emergency department before being transferred to the intensive care unit in private hospitals than in public hospitals [2 (1 - 3) *versus* 5 (2 - 24) hours; p < 0.001].

**Conclusion:**

This survey revealed significant heterogeneity in the organization of hospital resources, clinical practices and treatments among physicians during the early COVID-19 pandemic in Brazil.

## INTRODUCTION

Severe acute respiratory syndrome coronavirus 2 (SARS-CoV-2) has infected more than one hundred seventy million patients and caused more than four million deaths around the world as of August 2021.^(1)^ By the end of May 2020, Brazil became the epicenter of coronavirus disease 2019 (COVID-19).^(2)^ To date, more than 20 million Brazilians have been infected with SARS-CoV-2, while more than a half million deaths have been registered.^(3)^

Patients who require hospitalization have a high risk of developing acute respiratory distress syndrome (ARDS) and require admission to the intensive care unit (ICU) for organ support.^(4)^ Nevertheless, the COVID-19 pandemic has imposed an enormous strain on health care systems worldwide.^(5-10)^ Thus, the demand for ICU beds has been markedly supplanted by the capacity to care for patients.

Brazil’s health care system is characterized by important heterogeneity in terms of clinical practices and resource availability in public and private systems and within different regions of the country.^(11-13)^ Such disparities might have been exacerbated during the COVID-19 pandemic due to different organizational policies that were established to deal with the pandemic.

Information about organizational factors, clinical practices and the availability of resources in Emergency Departments (EDs) and ICUs in different regions of Brazil during the early months of the COVID-19 pandemic is limited. Therefore, we conducted a nationwide survey to address organizational, epidemiological and clinical aspects in Brazilian ICUs and EDs during the COVID-19 pandemic.

## METHODS

This was an observational, cross-sectional, nationwide survey reported in accordance with the A ConsensusBased Checklist for Reporting of Survey Studies (CROSS) statement.^(14)^

The questionnaire was a web-based survey with 49 questions divided into five main sections: demographics, structural organization for the pandemic, personal protection equipment (PPE), protocols and treatments.

The questions were initially structured by three specialists in emergency care and intensive care. The first draft of the survey was tested among all authors of this paper using the Delphi method; the authors were encouraged to make comments, modify the items, or propose new questions to be included in the questionnaire. A consequent version was obtained by consensus among all authors and sent to eight doctors, including intensivists and emergency physicians, to test the questionnaire and check for technical and comprehensive problems. Then, the final version of the questionnaire was constructed after discussion with agreement from all authors. The questionnaire is provided in full in Supplementary material (Appendix 1).

### Sample characteristics

This survey was provided to medical doctors from EDs and ICUs, whether they were specialists or not, who were treating patients infected with COVID-19 from May 30 to June 30, 2020. There was no restriction for response, and the only exclusion criterion was refusal to participate in the study.

The authors determined the sample size of 300 medical doctors based on the sample size reached by a previous Brazilian nationwide survey.^(15)^

### Survey administration

The electronic survey was supported and published online on the websites of *Associação de Medicina Intensiva Brasileira* (AMIB) and *Associação Brasileira de Medicina de Emergência* (ABRAMEDE); therefore, physicians from all regions of Brazil could access the online survey on the websites mentioned above and participate in the study. To increase participant recruitment, the authors published the survey link on their social media accounts. Research Electronic Data Capture (REDCap), an electronic system for data collection and management for scientific research,^(16)^ was used for the survey application and data extraction.

### Ethical considerations

The questionnaire cover letter informed the participants that participation in the study was completely voluntary and that their identities were *confidential*. When the participant proceeded to the questionnaire, their individual consent was implied, authorizing the use of the data.

The study was approved by the local Ethics Committee at *Hospita****l*** *Israelita Albert Einstein* with a waiver of informed consent (CAAE: 31484420.4.0000.0071).

### Statistical analysis

Participants were stratified according to the type of hospital in which they worked (public *versus* private hospitals), their work setting (ED *versus* ICU) and their geographic region (North, Northeast, Central-West, Southeast and South).

Categorical variables are presented as absolute and relative frequencies. Continuous variables are presented as medians with interquartile ranges (IQRs). Comparisons were performed between the pooled groups. Categorical variables were compared with the Chisquared test. Continuous variables were compared using the independent *t* test or Mann-Whitney U test in cases of nonnormal distribution, tested by the KolmogorovSmirnov test. All analyses were performed using R (R, version 3.6.0, Core Team, Vienna, Austria, 2016) software, and a two-sided alpha level of 0.05 was considered. Only complete surveys were included in the final analysis.

## RESULTS

Out of 351 participants who answered the survey, 67 participants with incomplete questionnaires were excluded. Two hundred eighty-four participants completed the questionnaire and were included in the final analysis.

### Characteristics of the survey participants and of the hospitals in which they worked

The characteristics of the survey participants and of the hospitals in which they worked, stratified by public or private hospitals, are described in table 1. The median age (IQR) was 39 years (33 - 47 years), 43.7% were female, 53.5% worked in public hospitals and 56% were from Southeast Brazil, the region in with the highest population density in Brazil. The most common medical specialty of the participants was intensive care (33.1%); only 8.5% were emergency specialists, 38.4% had other specialties and 20% were general practitioners.

**Table 1:** Characteristics of the survey participants and of the hospitals in which they worked (private ***versus*** public hospitals)

	All participants (n = 284)	Private hospitals (n = 132)	Public hospitals (n = 152)	p value*
Age (years)	39 (33 - 47)	40 (34 - 47)	39 (32 - 46)	0.148†
Female sex	124 (43.7)	53 (59.8)	71 (53.3)	0.321‡
Region				0.001‡
Central - West	17 (6.0)	3 (2.3)	14 (9.2)	
Northeast	38 (13.4)	16 (12.1)	22 (14.5)	
North	17 (6.0)	2 (1.5)	15 (9.9)	
Southeast	159 (56.0)	86 (65.2)	73 (48.0)	
South	53 (18.7)	25 (18.9)	28 (18.4)	
Medical specialty of the participants				< 0.001‡
Intensive care specialist	94 (33.1)	59 (44.7)	35 (23.0)	
Emergency medicine specialist	24 (8.5)	8 (6.1)	16 (10.5)	
General practitioner	57 (20.1)	15 (11.4)	42 (27.6)	
Other specialties	109 (38.4)	50 (37.9)	59 (38.8)	
Work in the ICU	160 (56.3)	83 (62.9)	77 (50.7)	0.051‡
NIV available	229 (80.6)	118 (89.4)	111 (73.0)	0.001‡
HFNC available	114 (40.1)	65 (49.2)	49 (32.2)	0.005‡
Video laryngoscope available	114 (40.1)	80 (60.6)	34 (22.4)	< 0.001‡
Number of ICU beds with negative pressure	2.0 (0.0 - 5.0)	2.0 (0.0 - 5.5)	0.0 (0.0 - 5.0)	0.011§
Average time (hours) to transfer a patient from the ED to the ICU	3.0 (1.0 - 6.0)	2.0 (1.0 - 3.0)	5.0 (2.0 - 24.0)	< 0.001§

ICU - intensive care unit; ED - emergency department; NIV - noninvasive ventilation; HFNC - high flow nasal cannula.

* p values were calculated with the use of the † independent t test; ‡ Chi-squared test; § Mann-Whitney U test. Values expressed as median (interquartile range) or n (%)

Comparing the private and public hospitals, most of the intensive care specialists who answered the survey worked in private centers. According to the participants, the number of ICU beds with negative pressure was higher in private hospitals. The responders estimated that patients spent 2 hours (1 - 3 hours) in the ED before transfer to the ICU in private hospitals, while in public hospitals, the surveyed physicians estimated that this transfer could take 5 hours (2 - 24 hours), with a significant difference (p < 0.001) (Table 1).

The participants answered that noninvasive ventilation (NIV) was available in 80% of the hospitals and high flow nasal cannula (HFNC) was available in only 40% of the hospitals. Noninvasive ventilation, HFNC and video laryngoscopy were more available in private hospitals than in public hospitals, with a significant difference (Table 1). According to the responders, the estimated proportion of COVID-19 patients using mechanical ventilation (MV) in the ICUs in their last shifts was statistically higher in public hospitals than in private hospitals (70% *versus* 50%) (Table 2).

**Table 2:** Proportion of COVID-19 patients (% of the total number of patients in the intensive care unit) who were in the intensive care unit and required advance life support, according to the participants who worked in intensive care units on their last shifts

	All participants (n = 160)	Private hospitals (n = 83)	Public hospitals (n = 77)	p value*
Mechanical ventilation	60 (15 - 80)	50 (15 - 73)	70 (20 - 80)	0.024†
Neuromuscular blockage	20 (1 - 40)	20 (2 - 40)	25 (1 - 50)	0.256†
Prone position	7 (0 - 20)	6 (0 - 10)	10 (0 - 20)	0.659†
Renal replacement therapy	25 (3 - 50)	20 (3 - 50)	30 (5 - 50)	0.475†
Vasoactive drugs	37.5 (10 - 66)	30 (5 - 50)	40 (15 - 70)	0.158†

* p values were calculated with the use of the (†) Mann-Whitney U test. Values expressed as median (interquartile range).

### Personal protection equipment

The responders’ answers about PPE availability are described in table 3. There was a significant difference regarding PPE availability between the different types of hospitals. All types of PPE were more accessible in private hospitals than in public hospitals according to the surveyed physicians, especially when comparing access to N95 or FFP masks. Most of the participants (87.7%) reported that they had a specific protocol for PPE utilization during tracheal intubation (Table 4).

**Table 3:** Questions about individual protection equipment recommended for treating COVID-19 patients

	All participants (n = 284)	Private hospitals (n = 132)	Public hospitals (n = 152)	p value*
Disposable gown				0.001†
Yes, and is never missing	196 (69.0)	106 (80.3)	90 (59.2)	
Yes, but is rarely missing	60 (21.1)	20 (15.2)	40 (26.3)	
Yes, but is frequently missing	19 (6.7)	5 (3.8)	14 (9.2)	
Not recommended	9 (3.2)	1 (0.8)	8 (5.3)	
Cap				0.002†
Yes, and is never missing	237 (83.5)	122 (92.4)	115 (75.7)	
Yes, but is rarely missing	33 (11.6)	6 (4.5)	27 (17.8)	
Yes, but is frequently missing	8 (2.8)	3 (2.3)	5 (3.3)	
Not recommended	6 (2.1)	1 (0.8)	5 (3.3)	
N95 or FPP mask				< 0.001†
Yes, and is never missing	206 (72.5)	113 (85.6)	93 (61.2)	
Yes, but is rarely missing	47 (16.5)	12 (9.1)	35 (23.0)	
Yes, but is frequently missing	25 (8.8)	4 (3.0)	21 (13.8)	
Not recommended	6 (2.1)	3 (2.3)	3 (2.0)	

* p values were calculated with the use of the (†) Chi-squared test. Values expressed as n (%).

**Table 4:** Questions about protocols and hospital organization during the COVID-19 pandemic

	All participants (n = 284)	Private hospitals (n = 132)	Public hospitals (n = 152)	p value*
Did your hospital increase the number of ICU beds during the pandemic?				0.097†
Yes	122 (76.2)	69 (83.1)	53 (68.8)	
No	36 (22.5)	13 (15.7)	23 (29.9)	
I do not know	2 (1.2)	1 (1.2)	1 (1.3)	
Does your hospital have a contingency plan for ICU shifts in case of medical license for COVID-19 infection?				< 0.001†
Yes	132 (46.5)	79 (59.8)	53 (34.9)	
No	113 (39.8)	37 (28.0)	76 (50.0)	
I do not know	39 (13.7)	16 (12.1)	23 (15.1)	
Does the ED of your hospital have a separate sector for patients with respiratory symptoms?				0.041†
Yes	253 (89.1)	122 (92.4)	131 (86.2)	
No	25 (8.8)	6 (4.5)	19 (12.5)	
I do not know	6 (2.1)	4 (3.0)	2 (1.3)	
Does a specific protocol for PPE utilization during tracheal intubation exist? Yes	249 (87.7)	124 (93.9)	125 (82.2)	0.005†
Does a specific protocol for attending COVID-19 patients exist?				0.322†
Yes	253 (89.1)	121 (91.7)	132 (86.8)	
No	26 (9.2)	10 (7.6)	16 (10.5)	
I do not know	5 (1.8)	1 (0.8)	4 (2.6)	
Does a specific protocol with well-established criteria for ICU admission for COVID-19 patients exist?				< 0.001†
Yes	218 (76.8)	115 (87.1)	103 (67.8)	
No	56 (19.7)	13 (9.8)	43 (28.3)	
I do not know	10 (3.5)	4 (3.0)	6 (3.9)	
Is there a sedation protocol for tracheal intubation for COVID-19 patients?				0.168†
Yes	216 (76.1)	106 (80.3)	110 (72.4)	
No	61 (21.5)	22 (16.7)	39 (25.7)	
I do not know	7 (2.5)	4 (3.0)	3 (2.0)	
Is there an invasive mechanical ventilation protocol for COVID-19 patients				0.006†
Yes	196 (69.0)	99 (75.0)	97 (63.8)	
No	77 (27.1)	25 (18.9)	52 (34.2)	
I do not know	11 (3.9)	8 (6.1)	3 (2.0)	
Is there a sedation protocol for mechanical ventilation for COVID-19 patients?				0.057†
Yes	177 (62.3)	87 (65.9)	90 (59.2)	
No	94 (33.1)	36 (27.3)	58 (38.2)	
I do not know	13 (4.6)	9 (6.8)	4 (2.6)	
Is there a protocol for the use of neuromuscular blockage in COVID-19 patients?				0.011†
Yes	165 (58.1)	85 (64.4)	80 (52.6)	
No	102 (35.9)	36 (27.3)	66 (43.4)	
I do not know	17 (6.0)	11 (8.3)	6 (3.9)	
Is there a specific protocol for weaning COVID-19 patients from mechanical ventilation?				0.002†
Yes	122 (43.0)	71 (53.8)	51 (33.6)	
No	115 (40.5)	41 (31.1)	74 (48.7)	
I do not know	47 (16.5)	20 (15.2)	27 (17.8)	
Did any COVID-19 patient at your ICU use ECMO?				< 0.001†
No, I do not have ECMO in my hospital	113 (70.6)	43 (51.8)	70 (90.9)	
No, I have ECMO in my hospital but did not use this therapy for COVID-19 patients	25 (15.6)	20 (24.1)	5 (6.5)	
Yes	22 (13.8)	20 (24.1)	2 (2.6)	

ICU - intensive care unit; ED - emergency department; PPE - personal protection equipment; ECMO - extracorporeal membrane oxygenation.

* p values were calculated with the use of the (†) Chi-square test. Values expressed as n (%).

### COVID-19 protocols and treatments

Most of the hospitals where the participants worked (89.1%) had a specific protocol for assisting COVID-19 patients, and 76.8% had a protocol with well-established criteria for ICU admission; however, according to the surveyed physicians, all types of protocols were more common in private hospitals than in public hospitals (Table 4).

Corticosteroids were the most common specific treatment prescribed, followed by chloroquine or hydroxychloroquine with macrolides in 57% and 35.9%, respectively, according to the surveyed physicians. Private hospitals prescribed more chloroquine or hydroxychloroquine with macrolides and more interleukin 6 inhibitors (Supplementary material - Table 1S).

Deep venous thrombosis (DVT) prophylaxis for COVID-19 patients (Supplementary material - Table 1S) was prescribed by almost all the participants, and the most common strategy reported to be prescribed was 40 mg of enoxaparin once a day.

More than half of the participants (64.1%) changed DVT prophylaxis for COVID-19 patients with elevated D-dimer levels, but different strategies were chosen (Supplementary material - Table 1S). Among the surveyed participants who changed their anticoagulation strategy according to D-dimer levels, 33.5% answered that there was no consensus about which D-dimer value they should use to change the treatment strategy. Extracorporeal membrane oxygenation (ECMO) was more available in private centers (Supplementary material - Table 1S).

Comparing the EDs and ICUs (Supplementary material - Table 2S), the latter had significantly more frequent adoption of protocols regarding how to sedate, ventilate, and intubate and the use of neuromuscular blockage, according to the surveyed physicians. Only 50% of the participants answered that they followed a specific protocol for weaning COVID-19 patients from MV, and it was less frequent in the EDs.

When treating mechanically ventilated patients, there was a significant difference between how positive endexpiratory pressure (PEEP) was set by doctors working in the EDs and ICUs. In the EDs, the most common way to set PEEP was according to oxygen saturation (SaO_2_ or SpO_2_), while in the ICUs, it was according to best compliance. In the EDs, 25% of the respondents did not know how to set PEEP (Supplementary material - Table 2S).

### Intensive care unit organization and resources stratified by Brazilian regions

The majority of the responders who worked in ICUs (77.5%) reported that their workplace increased the number of ICU beds for COVID-19 patients; however, only 37.5% of the responders from the Central-West region of Brazil reported an increase in ICU beds. NIV and video laryngoscopy were more available in the Southeast region and the HNFC in the Central-West region of Brazil (Supplementary material - Table 3S), according to the surveyed physicians.

## DISCUSSION

### Limitations

This study had several limitations, such as the large number of participants who did not complete the survey and the heterogeneity among the number of participants per region, which may not reflect the reality around the country. An important limitation was that all data were based on surveyed physicians’ subjective perceptions of care and did not include data collected from patients; therefore, all results need to be carefully evaluated for high risk or participant bias. Moreover, some answers to our survey, especially those concerning treatment, were time-related to when the survey was applied (first wave), and the answers could now be different, especially in clinical management regarding the beliefs of the benefit of early intubation, the safety of health care professionals and other patients regarding aerosol production with the use of NIV and HFNC, and the use of hydroxychloroquine and macrolides. These beliefs might have affected the development of the questionnaire. Even with COVID-19 treatment variations over time, the survey showed high heterogeneity among physicians’ practices. Finally, we did not ask about the time of initiation and dose of different pharmacological treatments included in this survey.

### Interpretations

Our results demonstrated significant differences in health care between public and private hospitals, between ICUs and EDs and in different regions of the country. Brazil has its own characteristics, such as regional economic differences (states in the South and Southeast have a higher socioeconomic index), better hospital structure, and the availability of ICU beds and intensivists.^(17)^

Data from our survey suggested some organizational, equipment, training and human resource limitations. First, there was difficulty in accessing ICU beds, reflected by a median ED wait time of 5 hours for a vacancy, based on the surveyed physicians’ perceptions. A longer stay in the ED might be detrimental to patient care, since protocols for sedation, tracheal intubation, MV, neuromuscular blockage and MV weaning are more frequent in the ICU, according to our survey. Similarly, it has been shown that patients on MV who stay in the ED for a longer period of time while waiting for an ICU bed have a worse prognosis.^(18)^

Second, the unavailability of NIV and HFNC, important resources for the treatment of COVID-19 patients,^(19)^ and the difficulty of ventilating patients, either due to the lack of access to items such as neuromuscular blockers or the lack of knowledge of the team regarding the choice of PEEP, are much greater in EDs. Third, emergency medicine was only recognized as a medical specialty in Brazil in 2015; therefore, there is a reduced number of specialists in the EDs in the country.^(20)^ The Brazilian national ICU registry, *UTIs Brasileiras - Registro Nacional de Terapia Intensiva* (www.utisbrasileiras.com.br), reveals an important difference in hospital mortality for COVID-19 patients when comparing public *versus* private hospitals (51% *versus* 28%).^(21)^ The organizational, technical, equipment and human resource aspect differences between public *versus* private hospitals, according to our survey, might contribute to a higher number of deaths in public centers. Nonetheless, we need to consider that the worst outcomes in public hospitals might also be related to understaffing and a low number of available ICU beds, forcing physicians to admit sicker patients in the ICU and causing a selection bias of the type of critically ill patients arriving in public ICUs later when compared to private ICUs; these patients have more multiple organ failure evolution since they were first admitted to a primary care hospital, transferred to the ED and finally transferred to the ICU, which is a process that can last for days.

During this pandemic, concerns about the safety of health care professionals with adequate PPE and medical devices such as videolaryngoscopy have been a cornerstone in preventing COVID-19 contamination.^(22)^ Official authorities reported 81,574 notified COVID-19 flu syndrome cases in Brazilian health care professionals as of April 19, 2021.^(23)^ Our results showed that public hospitals sometimes run out of N95 or FPP masks (public 14% *versus* private hospitals 3%) and disposable gowns (9% *versus* 4%, respectively). Moreover, this research reported that items that can help with patient management, such as the videolaryngoscope, were often unavailable.^(24)^

Severe COVID-19 patients require highly complex treatment and well-trained professionals. Data collected from the surveyed physicians showed that in their last shifts, 60% of the patients in the ICU were mechanically ventilated, of which 20% were on neuromuscular blockades and only 7% were in the prone position. One-quarter of the patients were on renal replacement therapy, and one-third were on vasoactive drugs. Our results showed that COVID-19 patients in Brazilian ICUs might have lower rates of MV than those reported in international data, in which the reported rates ranged from 50% to 100%.^(25-27)^ This might be explained by the differences in the COVID-19 pandemic timeline in Europe, Asia and North America compared to Brazil. Different data in Brazil were found in a recent cohort study. In this study, 79% of the patients in the ICU received MV, with 16% of these patients in the prone position.^(25)^ Another cohort with 13301 patients reported the need for ventilatory support in 31% of the patients, of which 42% required invasive MV.(26)

When stratified by type of hospital, public hospitals had more mechanically ventilated patients in the ICU than private hospitals (70% *versus* 50%; p = 0.024), according to the surveyed physicians. Factors that can be associated with this difference are a longer time spent in the ED waiting for an ICU bed, where intensive care is sometimes not adequate, lower access to NIV and HFNC and lower numbers of treatment protocols. Our survey showed estimates that Brazilian critically ill COVID-19 patients in the ICU were less frequently in the prone position and on vasoactive drugs than those in previous international studies reporting up to 47% and 66%, respectively.^(27-29)^ Data on the use of neuromuscular blockage and renal replacement therapy are similar to ours.^(27-29)^ Most likely, the reason for lower rates of prone positioning in public hospitals is the necessity of a high number of trained professionals required to perform this maneuver, and this is not the reality in some hospitals in Brazil.

Extracorporeal membrane oxygenation is a rescue therapy for severe ARDS that is refractory to conventional management and can be lifesaving.^(30,31)^ International studies have revealed that up to 5% of critically ill COVID-19 patients require ECMO support.^(28,29)^ Our survey showed that ECMO is rarely available in Brazil, and 70% of hospitals do not have this type of support, according to the surveyed physicians. When stratified by type of hospital, the scarcity of this resource was even greater; 91% of public hospitals *versus* 52% of private hospitals do not have ECMO. Therefore, rescue therapy for refractory acute respiratory failure with extracorporeal support is not the reality in Brazil and is available in few centers.

Regarding COVID-19 pharmacological treatment, the most frequent medication prescribed was corticosteroids, but it was only prescribed by 57% of the participants. Chloroquine or hydroxychloroquine plus macrolides was the second most frequently prescribed drug. In private hospitals, compared to public hospitals, there was a higher rate of prescription of chloroquine or hydroxychloroquine plus macrolides (30% *versus* 43%; p = 0.024) and interleukin-6 inhibitors (2.6 *versus* 11.4%; p = 0.007), according to the surveyed physicians. By the time of this survey, there were no robust published data showing survival benefits with the administration of corticosteroids in severe COVID-19 patients^(32,33)^ and no potential harmful or beneficial clinical effects concerning chloroquine or hydroxychloroquine.^(34,35)^ Thus, the results of pharmacological treatment might not reflect actual practice.

COVID-19 infection is known to increase thromboembolic events. Therefore, anticoagulants are essential to prevent complications.^(36)^ This survey showed that almost all Brazilian doctors (96%) prescribe DVT prophylaxis. However, which medication should be prescribed and the adequate dosage are still under debate worldwide.^(36)^

Our research has strengths. To date, this is the first nationwide COVID-19 survey from Latin America regarding issues of structural, epidemiological and clinical aspects of the pandemic in Brazil. Our study included participants from all regions of the country, public and private health care systems, and EDs and ICUs, resulting in a more reliable overview of different sectors of the Brazilian health care system.

## CONCLUSION

This survey revealed significant heterogeneity in the organization of hospital resources, clinical practices and treatments among physicians across the country. This heterogeneity might have an impact on the outcomes of patients.
